# Comparison of Transcanalicular Multidiode Laser Dacryocystorhinostomy with and without Silicon Tube Intubation

**DOI:** 10.1155/2016/6719529

**Published:** 2016-03-31

**Authors:** Yildiray Yildirim, Taner Kar, Tuncay Topal, Enver Cesmeci, Abdullah Kaya, Kadir Colakoglu, Yakup Aksoy, Murat Sonmez

**Affiliations:** ^1^Department of Ophthalmology, Gulhane Military Medical Academy Haydarpasa Training Hospital, Istanbul, Turkey; ^2^Department of Otorhinolaryngology, Diyarbakir Military Hospital, Diyarbakir, Turkey; ^3^Department of Ophthalmology, Anittepe Military Dispensary, Ankara, Turkey; ^4^Department of Ophthalmology, Kasimpasa Military Hospital, Istanbul, Turkey; ^5^Department of Ophthalmology, Girne Military Hospital, Girne, Northern Cyprus, Mersin 10, Turkey

## Abstract

*Aim*. To compare the surgical outcomes of surgery with and without bicanalicular silicon tube intubation for the treatment of patients who have primary uncomplicated nasolacrimal duct obstruction.* Methods*. This retrospective study is comprised of 113 patients with uncomplicated primary nasolacrimal duct obstruction. There were 2 groups in the study: Group 1 (*n* = 58) patients underwent transcanalicular diode laser dacryocystorhinostomy surgery with bicanalicular silicon tube intubation and Group 2 (*n* = 55) patients underwent transcanalicular diode laser dacryocystorhinostomy surgery without bicanalicular silicon tube intubation. The follow-up period was 18.42 ± 2.8 months for Group 1 and 18.8 ± 2.1 months for Group 2.* Results*. Success was defined by irrigation of the lacrimal system without regurgitation and by the absence of epiphora. Success rates were 84.4% for Group 1 and 63.6% for Group 2 (*P* = 0.011). Statistically a significant difference was found between the two groups.* Conclusion*. The results of the study showed that transcanalicular diode laser dacryocystorhinostomy surgery with bicanalicular silicon tube intubation was more successful than the other method of surgery. Consequently, the application of silicone tube intubation in transcanalicular diode laser dacryocystorhinostomy surgery is recommended.

## 1. Introduction

Nasolacrimal duct obstruction (NLDO) is the most common cause of chronic dacryocystitis and in this case the only treatment option is surgery [1, 2]. Although the external surgical approach still is the gold standard with the highest success rate, the most recent stage in the development of dacryocystorhinostomy (DCR) is the endocanalicular or transcanalicular approach. In this approach, a probe with a red light on the end is inserted outside punctum and is moved toward the nasal wall [3]. Subsequently, nasal osteotomy is performed by diode laser energy [4].

Laser-assisted DCR application began with Massaro et al. in 1990; and, in addition to argon laser diode, potassium titanyl phosphate (KTP), holmium YAG, CO_2_, Nd:YAG, and erbium lasers have also been used until today [5–7]. New ostium is created by these lasers from an intranasal or transcanalicular approach.

Flexible endoscopes (0.3–0.7 mm diameter) modified from gastroduodenal endoscopes were developed for transcanalicular surgery [8]. By extending the diameter of the endoscopes and increasing the pixels of imaging, better quality results have been obtained.

Some authors have suggested the use of silicon tube intubation in NLDO surgery [9, 10], while some prefer using silicon tubes only for definitive indications (canalicular damage, lacrimal sac inflammation, secondary surgery, small and contracted sacs, etc.) [11, 12].

The purpose of this retrospective study was to compare the surgical outcomes of transcanalicular diode laser dacryocystorhinostomy (TDL-DCR) surgery with and without bicanalicular silicon tube intubation in the treatment of a series of 113 patients with primary uncomplicated nasolacrimal duct obstruction.

## 2. Subjects and Methods 

### 2.1. Subjects

A series of 113 patients who had not previously undergone this surgery were operated on for NLDO between 2010 and 2013. The study was carried out in accordance with the tenets of the Declaration of Helsinki. Approval for the study was granted by the Clinical Research Ethics Committee of GATA Haydarpasa Training Hospital (1491-59-14/1539) and informed consent was obtained from all the patients. A retrospective review was made of the 2 groups of patients. Group 1 is comprised of 58 patients who underwent TDL-DCR surgery with bicanalicular silicon tube intubation, and Group 2 is comprised of 55 patients who underwent TDL-DCR surgery without bicanalicular silicon tube intubation.

After complete ophthalmic examination, nasolacrimal duct obstruction was confirmed with lacrimal irrigation and dacryocystography with Lipiodol® preoperatively in each case. Blood tests were obtained from all patients to analyze systemic diseases.

The patients were selected according to the following criteria: (i) no history of nasolacrimal duct surgery; (ii) no canalicular obstruction; (iii) no history of traumatic injury to the ocular or nasal region; (iv) no concomitant nasal pathology, such as septum deviation, concha bullosa, nasal polyposis, and atrophic rhinitis; (v) absence of active infective dacryocystitis; (vi) absence of dry eye and lower lid laxity.

### 2.2. Methods

All operations were performed as in another previous study [13]. All operations were performed under local anesthesia. Before the surgery, topical anesthetic drops (oxybuprocaine hydrochloride 0.4%) were put on the conjunctiva and cornea. Then intranasal, infraorbital, and lateral nasal side anesthesia were applied with a solution mixture of epinephrine hydrochloride and lidocaine.

**Figure 1:**
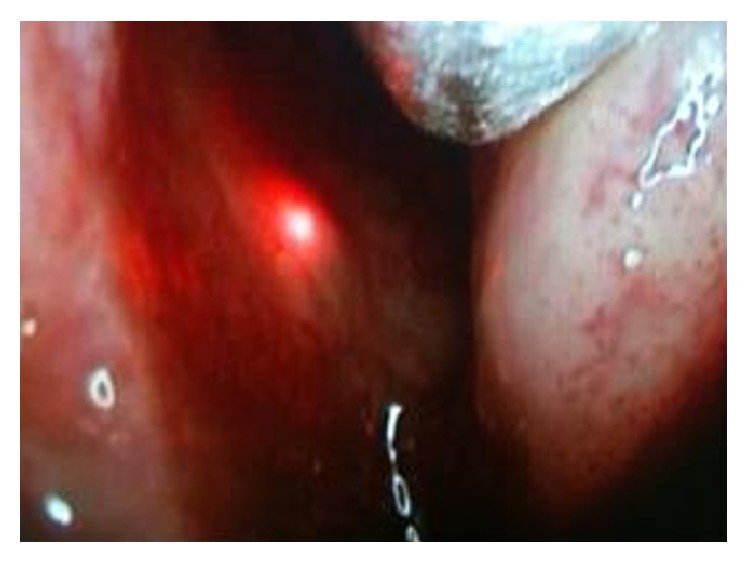
The red light reflex of the fiber is clearly seen on the nasal wall of the middle turbinate.

After dilating the lacrimal puncta, the fiber was inserted through the canaliculus to the wall of the sac. The feeling of a hard stop is essential during the insertion process. With the endoscopic visualization of the nasal cavity, the red light reflex of the fiber is clearly seen on the nasal wall of the middle turbinate plane (Figure 1). In this way, the target tissue was determined by the laser light guide.

**Figure 2:**
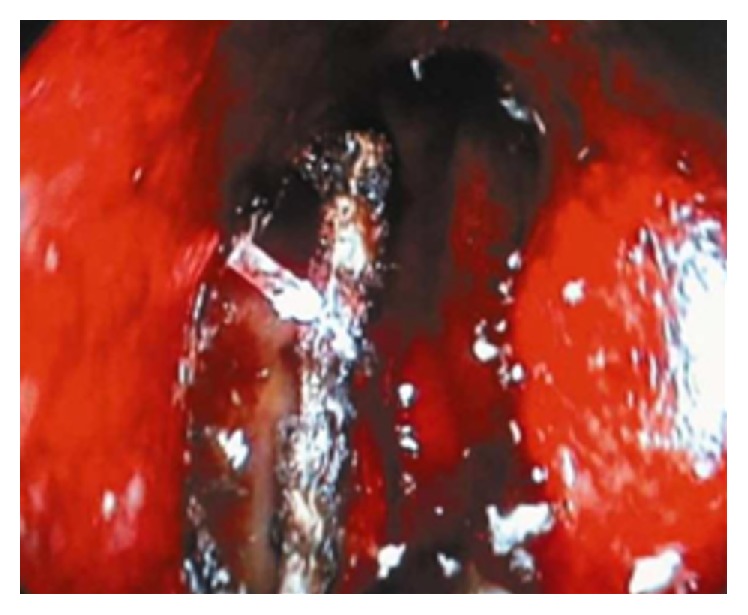
The ostium was expanded sufficiently with the fiber manipulation.

Diode laser (INTERmedic*™* diode S30 OFT 980 nm) parameters were settled at 10 W in 500 ms pulse mode potency, taking care not to prolong each impact to avoid overheating the structures. After reaching the nasal cavity, the osteotomy was expanded sufficiently with the fiber manipulation. A Crawford-type aspirator was used to displace the middle turbinate medially to protect the septum and middle turbinate and to maintain adequate exposure to the surgical site. Laser application was continued until the width of the new ostium becomes greater than 5 mm diameter (Figure 2). The size of osteotomy was controlled by the use of the nasal endoscope. The laser shots were between 28 and 45 shots at 10 watts. At the end of surgery, the laser probe was removed and lacrimal irrigation with saline solution was administered.

**Figure 3:**
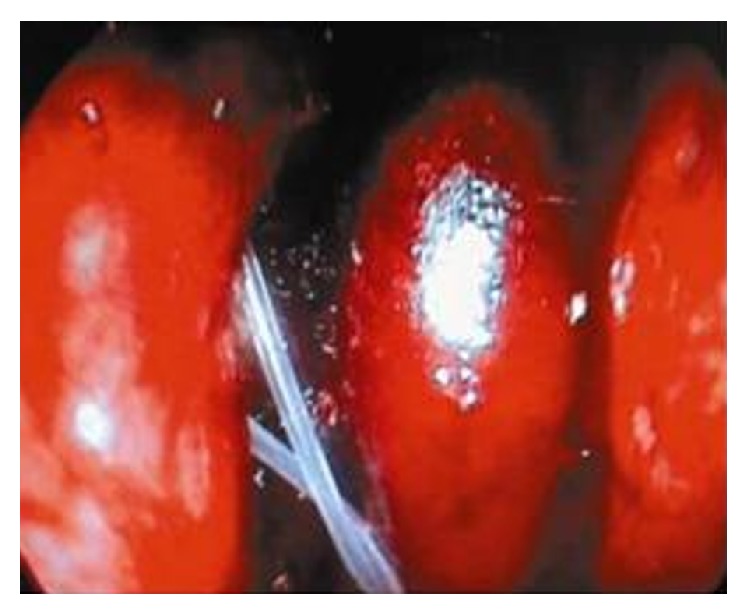
The silicon tubes were left free in the nasal cavity.

In addition to the surgery in Group 1 (*n* = 58), bicanalicular silicone intubation was performed. Silicone extensions of the tube were tied to each other and then were left free in the nasal cavity (Figure 3). Tamponade was applied to the nasal cavity to ensure control of the bleeding.

Postoperatively, antibiotic and steroid eye drops, nasal steroid spray, and also nasal saline were to be used four times a day for 2 weeks. Additionally, oral antibiotic was to be used for 7 days.

Follow-up postoperative examinations were carried out on the first day, in the first week, in the first month, in the 3rd month, and then at 3-month intervals. Silicone tube was removed 3 months after intubation. In follow-up visits, the patency of the lacrimal drainage system was checked. Resolution of symptomatic epiphora and lack of resistance in nasolacrimal saline irrigation were defined as success. The follow-up time was at least 12 months.

### 2.3. Statistical Analysis

Data analyses were performed using SPSS 14.0 (Statistical Package for Social Sciences, SPSS Inc., Chicago, IL). The normal distribution of the considered variables was first evaluated using the Shapiro-Wilk test. The data was presented as the mean ± standard deviation for the continuous variables, and the number of cases was used for the categorical ones. Independent samples *t*-test was used to compare the means between Group 1 and Group 2. The differences between the groups were analyzed by Chi-square tests. A value of *P* ≤ 0.05 was accepted as statistically significant.

## 3. Results

The study is comprised of 113 patients: Group 1 was composed of 58 patients (28 males, 30 females) with a mean age of 33.6 ± 11.57 (21–65) years and Group 2 was composed of 55 patients (21 males, 34 females) with a mean age of 37.4 ± 10.01 (21–65) years. Final success rates were (49/58) 84.4% for Group 1 and (35/55) 63.6% for Group 2 (*P* = 0.011). The mean surgical time for Groups 1 and 2 was 15.96 ± 3.01 and 13.74 ± 3.66 mins (range: 9–21 mins in both groups), respectively. The mean surgical time was longer due to silicon tube tying in Group 1 and there was a statistically significant difference among the groups (*P* = 0.001). The mean total laser energy of Groups 1 and 2 was 670.52 ± 49.18 and 651.09 ± 49.57 Joules (range: from 420 to 720 Joules in both groups), respectively. There was no statistically significant difference among the groups in terms of total laser energy (*P* = 0.951).

In Group 1, endoscopic examinations showed granulomas in 3 patients. These granulomas were removed by endoscopic procedures. In 6 cases, the result was evaluated as a failure, as there was mucosal scarring around the osteotomized area, and reobstruction occurred between 3 and 6 months postoperatively in 4 patients and between 6 and 12 months in 2 patients. In the 2nd month, 1 patient in Group 1 developed an episode of infection, which was immediately treated with medical therapy. Except for that patient, there were no other complications such as erosion of the punctum, fistulation to skin, and removal of the tubes. In Group 2, endoscopic examinations showed scarring of the internal ostium requiring secondary surgery in 20 of the patients. Reobstruction occurred between 1 and 3 months in 12 patients, between 3 and 6 months in 6 patients, and between 6 and 12 months in 2 patients postoperatively. The follow-up period was 18.4 ± 2.8 months for Group 1 and 18.8 ± 2.1 months for Group 2.

## 4. Discussion

Transcanalicular diode laser dacryocystorhinostomy (TDL-DCR) is a minimally invasive surgical procedure, which has the great advantage of accessing the operating field through anatomic pathways. It minimizes trauma to surrounding tissue, avoids unnecessary surgical skin scars, and provides precise cutting and removal of tissue by ablation. In addition, TDL-DCR causes minimum pain and minimum nasal bleeding. It is also easier and faster to perform compared to the classical dacryocystorhinostomy. Silicon tube intubation with DCR surgery is used to prevent the blocking of the lacrimal passage and to provide epithelization. Since silicon is an inert substance, it does not damage the conjunctiva and can be well-tolerated in the canaliculi.

As mentioned above, the use of silicon tube intubation has been suggested for patients with coexisting canalicular diseases, contracted or scarred lacrimal sacs, and persistent congenital nasolacrimal duct obstructions. Allen et al. [14] evaluated 242 cases retrospectively and showed no statistically significant difference between failure and age but a statistically significant difference between failure and silicon tube intubation. In their study, it was reported that formation of granulomatous tissue at the site of osteotomy is one of the most important failure factors in surgery with silicon tube intubation.

In literature, there are few studies about DCR surgery with and without silicon tubes. While some studies have reported no statistically significant advantage of using DCR with silicon stents over the DCR without stents [15–17], in the other studies, intubation is recommended in DCR surgery [9].

Feng et al. [16] concluded that no benefit was found in silicon tube intubation in primary DCR based on a meta-analysis of primary dacryocystorhinostomy with and without silicon intubation that included 9 trials involving 514 cases.

In the current study, the TDL-DCR surgery group with silicon tubes had a success rate of 84.4% (49/58), while the other group without tubes had a success rate of 63.6% (35/55), with a significant difference between these groups (*P* < 0.05). The success rates of both groups in the current study were similar to previous reports. Success rates have been reported to vary between 80% and 99% in external DCR surgery and between 58% and 97% in endoscopic nasal procedures [18–22]. The success of this combination with silicon tubes in TDL-DCR, possibly occurring ostium closure, is due to inhibition by the silicon tube during wound healing.

There were a total of 29 failures in this study: 9 in Group 1 and 20 in Group 2. Formation of granulomatous tissue occurred in 3 failed cases in Group 1. In this group, dacryocystitis was also observed in 1 patient. In addition, endoscopic examinations showed scarring of the internal ostium in 6 patients in Group 1 and in 20 patients in Group 2.

Rebeiz et al. [23] suggested 4 to 6 weeks for the duration of silicon tube intubation. To prevent the formation of granuloma, Kong et al. [24] suggested not removing the tubes before 8 weeks. Häusler and Caversaccio [25] reported that the tubes were well-tolerated by the patients and permit drainage of the nasolacrimal ducts for months and even years. In that study, the tubes remained in place for 9 months on average. In the current study, the silicon tubes were removed 3 months after surgery. The bicanalicular tubes in the lacrimal ducts were well-tolerated by all patients without notable problems except in 1 patient who developed an infection. The great number of female participants compared to the males was consistent with previous findings [18, 20].

This study concluded that the success rate was different in the two TDL-DCR surgery groups with and without silicon tubes. Silicon tube intubation was advantageous for patients who were undergoing their first dacryocystorhinostomy surgery for nasolacrimal duct obstruction. On the basis of these different outcomes, bicanalicular silicon tube intubation should be used in TDL-DCR surgery for patients with primary nasolacrimal duct obstruction.

## References

[1] Hartikainen J., Antila J., Varpula M., Puukka P., Seppä H., Grénman R. (1998). Prospective randomized comparison of endonasal endoscopic dacryocystorhinostomy and external dacryocystorhinostomy. *Laryngoscope*.

[2] Lee S., Yen M. T. (2011). Laser-assisted dacryocystorhinostomy: a viable treatment option?. *Current Opinion in Ophthalmology*.

[3] Parente Hernández B., Sentieri Omarrementería A., Junceda Moreno J. (2012). Corrective techniques of lacrimal obstruction in the vertical system. *Archivos de la Sociedad Espanola de Oftalmologia*.

[4] Strong E. B. (2013). Endoscopic dacryocystorhinostomy. *Craniomaxillofacial Trauma & Reconstruction*.

[5] Massaro B. M., Gonnering R. S., Harris G. J. (1990). Endonasal laser dacryocystorhinostomy. a new approach to nasolacrimal duct obstruction. *Archives of Ophthalmology*.

[6] Gonnering R. S., Lyon D. B., Fisher J. C. (1991). Endoscopic laser-assisted lacrimal surgery. *American Journal of Ophthalmology*.

[7] Woog J. J., Metson R., Puliafito C. A. (1993). Holmium: YAG endonasal laser dacryocystorhinostomy. *American Journal of Ophthalmology*.

[8] Emmerich K. H., Luchtenberg M., Meyer-Ruesenberg H. W., Steinhauer J. (1997). Dacryoendoscopy and laser dacryoplasty: technique and results. *Klinische Monatsblätter für Augenheilkunde*.

[9] Zuhair Al-Asadi S., Muhammed Al-Abbasi A. (2011). Efficacy of diode laser transcanalicular dacryocystorhinostomy in acquired nasolacrimal duct obstruction. *Gomal Journal of Medical Sciences*.

[10] Woog J. J., Kennedy R. H., Custer P. L., Kaltreider S. A., Meyer D. R., Camara J. G. (2001). Endonasal dacryocystorhinostomy: a report by the American Academy of ophthalmology. *Ophthalmology*.

[11] Doucet T. W., Hurwitz J. J. (1982). Canaliculodacryocystorhinostomy in the treatment of canalicular obstruction. *Archives of Ophthalmology*.

[12] Ari Ş., Cingü A. K., Şahin A., Gün R., Kiniş V., Çaça I. (2012). Outcomes of revision external dacryocystorhinostomy and nasal intubation by bicanalicular silicone tubing under endonasal endoscopic guidance. *International Journal of Ophthalmology*.

[13] Yildirim Y., Salihoglu M., Kar T. (2015). Postoperative changes in olfactory function after transcanalicular diode laser dacryocystorhinostomy. *Ophthalmic Plastic and Reconstructive Surgery*.

[14] Allen K., Berlin A. J. (1989). Dacryocystorhinostomy failure: association with nasolacrimal silicone intubation. *Ophthalmic Surgery*.

[15] Al-Qahtani A. S. (2012). Primary endoscopic dacryocystorhinostomy with or without silicone tubing: a prospective randomized study. *American Journal of Rhinology and Allergy*.

[16] Feng Y.-F., Cai J.-Q., Zhang J.-Y., Han X.-H. (2011). A meta-analysis of primary dacryocystorhinostomy with and without silicone intubation. *Canadian Journal of Ophthalmology*.

[17] Smirnov G., Tuomilehto H., Teräsvirta M., Nuutinen J., Seppä J. (2008). Silicone tubing is not necessary after primary endoscopic dacryocystorhinostomy: a prospective randomized study. *American Journal of Rhinology*.

[18] Henson R. D., Cruz H. L., Henson R. G., Ali M. J., Kakizaki H. (2012). Postoperative application of mitomycin-C in endocanalicular laser dacryocystorhinostomy. *Ophthalmic Plastic and Reconstructive Surgery*.

[19] McLachlan D. L., Shannon G. M., Flanagan J. C. (1980). Results of dacryocystorhinostomy: analysis of the reoperations. *Ophthalmic Surgery*.

[20] Pal V. K., Agrawal A., Suman S., Pratap V. B. (2013). Transcanalicular endoscope combined laser-assisted dacryocystorhinostomy. *Oman Journal of Ophthalmology*.

[21] Horix D., Struck H. G. (2004). Long term patency rate of the external dacryocystorhinostomy. A retrospective study in the years 1991–2000 at the University Eye Hospital in Halle. *Ophthalmologe*.

[22] Erdöl H., Akyol N., Imamoglu H. I., Sözen E. (2005). Long-term follow-up of external dacryocystorhinostomy and the factors affecting its success. *Orbit*.

[23] Rebeiz E. E., Shapshay S. M., Bowlds J. H., Pankratov M. M. (1992). Anatomic guidelines for dacryocystorhinostomy. *Laryngoscope*.

[24] Kong Y. T., Kim T. I., Kong B. W. (1994). A report of 131 cases of endoscopic laser lacrimal surgery. *Ophthalmology*.

[25] Häusler R., Caversaccio M. (1998). Microsurgical endonasal dacryocystorhinostomy with long term insertion of bicanalicular silicone tubes. *Archives of Otolaryngology—Head and Neck Surgery*.

